# Barriers to accessing adequate maternal care in Georgia: a qualitative study

**DOI:** 10.1186/s12913-018-3432-z

**Published:** 2018-08-13

**Authors:** Elina Miteniece, Milena Pavlova, Lela Shengelia, Bernd Rechel, Wim Groot

**Affiliations:** 1 Department of Health Services Research, Maastricht University, Faculty of Health, Medicine and Life Sciences, Duboisdomein 30, Maastricht, 6229 GT The Netherlands; 2 Top Institute Evidence-Based Education Research (TIER), Maastricht University, Kapoenstraat 2, 6211 KH Maastricht, The Netherlands; 3 European Observatory on Health Systems and Policies, London School of Hygiene & Tropical Medicine, Keppel Street, London, WC1E 7HT UK; 4 National Center for Disease Control and Public Health (NCDC) Georgia, Asatiani street 9, 0177 Tbilisi, Georgia

**Keywords:** Georgia, Central and Eastern Europe, Maternal care, Access, Pregnancy, Appropriateness, Approachability, Cost

## Abstract

**Background:**

The maternal health outcomes in Georgia are linked to shortcomings in healthcare such as inequities in access to adequate maternal care. Due to the macro-level, quantitative approach applied in most previous studies, little is known about the underlying reasons that influence maternal care and care-seeking behaviour of pregnant women.

**Methods:**

This qualitative study explores the stakeholders’ perspectives on access to adequate maternal care in Georgia. Focus-group discussions are conducted with mothers who gave birth within in the past four years and in-depth interviews are conducted with decision-makers and health professionals in the field. Five access-related aspects are studied: availability, appropriateness, affordability, approachability and acceptability of maternal care. The method of direct content analysis is applied.

**Results:**

Results indicate problems with maternal care standards, inequalities across population groups and drawbacks in maternal care financing. This includes gaps in clinical quality and staff skills, as well as poor communication between women and health professionals. Geographical barriers to adequate maternal care exist in rural and mountainous areas due to the weak infrastructure (poor roads and weak transportation), in addition to financial hardships. Despite improvements in the coverage of maternal care, affordability remains an access barrier. Poorer population groups are financially unprotected from the high out-of-pocket payments for maternal care services.

**Conclusion:**

These findings imply that micro-level indicators, such as disrespectful behaviour of health professionals and affordability of care, should be taken into account when assessing maternal care provision in Georgia. It should complement the existing macro-level indicators for a comprehensive evaluation of maternal care.

**Electronic supplementary material:**

The online version of this article (10.1186/s12913-018-3432-z) contains supplementary material, which is available to authorized users.

## Background

The official maternal mortality rate (MMR) in Georgia is high, at 31 deaths per 100,000 live births in 2014 according to clinical data [1]. The MMR in Georgia is higher than in neighbouring former Soviet Republics such as Armenia (19 per 100,000 in 2014) or Azerbaijan (15 per 100,000 in 2014) [1]. It is more than three times higher than the MMR average for the WHO European region (10 per 100,000 in 2014) and more than six times the European Union average (four per 100,000 in 2014) [1]. Yet, maternal mortality in Georgia might be even higher (with an estimated 36 deaths per 100,000 live births in 2015), as the official clinical or cause of death data undercount maternal mortality [2, 3].

The government has taken a number of steps to reduce maternal mortality and reach Millennium Development Goal 5 (MDG5), which aimed to reduce the MMR by three quarters between 1990 and 2015 [1, 4]. Yet, the MMR remains higher than desired under the MDG5. The previous MDG5 fits within the current aim 3.1 of the Sustainable Development Goals 2015–2030 to reduce the Global MMR. Achieving the desired MMR target by 2030 requires an annual reduction in MMR of at least 7.5%, which is double the progress of MDG5 [5]. Interventions to achieve this include staff retraining, infrastructure development, screening programs, and free-of-charge access to basic maternal care services [3].

The reasons for the high MMR in Georgia can be found at both the macro level (e.g. availability of infrastructure, facilities and medical staff) and the micro level (e.g. provision and use of services, communication, affordability, adequacy, awareness) [3, 6–8]. The high MMR in Georgia is also linked to inequities in access to adequate maternal care [3, 4]. Adequate care is understood here as care with good clinical quality that meets medical standards and is delivered in accordance with the preferences of service users (the pregnant women) [9].

Previous studies in Georgia have confirmed that barriers to access adequate maternal care services contribute to poor maternal health [3, 7, 10, 11]. Thus, the high maternal mortality risk is associated not only with physiological factors related to higher maternal age and poor pre-pregnancy health, but also with social factors, such as living in rural areas, low economic status and late care-seeking behaviour due to insufficient awareness or inability to pay. Quality of maternal care is another important aspect that causes a higher MMR risk [4, 12].

The Georgian maternal care system is rather complex and fragmented. The providers of maternal care include outpatient and inpatient maternity clinics in both the public and private sector, providing services within and outside the various state maternal care programs. The state programs are funded through annual state budget allocations and cover free-of-charge access to basic antenatal care (four visits), food supplements (folic acid), antenatal and maternal screening, management of high-risk pregnancies, and birthing care (including C-section). Outside the state programs, pregnant women may use additional maternal services that are paid by the state Universal Health Care program, private insurance schemes or out-of-pocket payments. Similar to such countries as Ukraine, maternal care in Georgia also seems to follow the so-called “technocratic model”, which stresses a “mind–body separation and sees the body as a machine” [13, 14]. According to this model, the obstetrician/gynaecologist is the key professional during the whole maternal period. Midwives have only a limited or no involvement in the maternal period. Midwives can be involved in childbirth but also then play only a secondary role, except when the obstetrician is not available. In line with the technocratic model of care, the structure of obstetric services differs from international standards of demedicalisation, and the goal to minimize interventions, avoid unnecessary interventions, provide “evidence-based care as well as intellectual, emotional, social, and cultural needs of women, their babies, and families” [15].

Although the use of antenatal care has increased since the state programs started funding four antenatal visits, about a quarter of pregnant women still do not receive such care in the first trimester and about 15% of pregnant women do not have at least four antenatal visits [16]. Furthermore, in 2010, around 2% of pregnant women, increasing to 5% among ethnic minority groups (mostly Armenians and Azeri), had a home childbirth without a skilled birth attendant [3]. This might help to explain why one fifth of maternal deaths occur among ethnic minorities. Potential reasons for the higher share of home childbirths among ethnic minorities include lower socio-economic status as well as insufficient access to maternal care [7].

According to the 2010 Reproductive Health Survey, the use of postnatal care in 2010 was only 23% and only a small share of women reported adequate counselling experience [3]. Moreover, gynaecological routine visits outside pregnancy remain low in Georgia (24%). Such visits are important contributors to the outcome of pregnancy, especially if there are gynaecological conditions present that negatively affect pregnancy [3, 7].

Another problem is the existence of high out-of-pocket payments for health services, which undermines access to care for the poorest population groups [4, 10]. Georgia stands out among the former Soviet Republics as the one with the highest share of private expenditures on health care [17], reaching 79% of total health expenditure in 2014 [1]. The 2010 Reproductive Health Survey found that 25% of pregnant women delayed the use of medical care, in the vast majority (82%) due to the high costs involved [3]. Despite the free-of-charge birthing care offered through the state maternal programs, maternal care providers (mostly the private establishments) frequently request extra out-of-pocket payments for the care received during birth [3, 10]. In this regard, problematic pregnancies are charged much higher by the providers, especially if the women need more than four antenatal visits and more complex childbirth care. Consequently, access to maternal care in Georgia is expensive and the state programs do not effectively protect women from this financial risk. Furthermore, there are substantial differences in the quality of care between providers and women report difficulties in accessing those providers they deem well-qualified [12].

In order to develop and implement well-designed policies and programs that address equity in maternal health access, it is necessary to understand the factors that generate and sustain barriers to maternal care use in Georgia [4]. Due to the macro-level, quantitative approach applied in most previous studies (some of those mentioned above), little is known about the underlying reasons and contextual factors that influence care-seeking behaviour of pregnant women in Georgia. Furthermore, since care-seeking behaviour is not only an outcome of individual decision-making, it should be investigated in the community, taking into account its cultural, social and political environment. This indicates the need for qualitative research to gain an in-depth understanding of access-related factors in maternal care in Georgia [7, 18].

The aim of our study is to explore stakeholder views on access to adequate maternal care in Georgia. We follow a qualitative research approach, based on data collected among women who have experienced childbirth within the last four years, providers of maternal care and decision-makers. This allows us to determine the extent of stakeholder consensus on barriers to access and underlying factors.

### Access to adequate maternal care – operational definitions

Levesque et al. distinguish five aspects of access to care, namely availability, appropriateness, affordability, approachability and acceptability [19]. A rather similar framework with slightly different aspects of access has been put forward by Obrist et al. and Putrik et al. among others [20, 21]. We apply the framework developed by Levesque et al. to study the barriers to accessing adequate maternal care in Georgia, operationalizing the five aspects of access in line with Levesque et al., as well as a review of recent literature on maternal care provision in Central and Eastern Europe [19, 22].

Availability reflects the geographical location, distribution and number of health care facilities, their opening hours, as well as the services and providers that the service users (childbearing women in our case) can choose from [19]. This means that access to maternal care could be limited in certain locations due to the unavailability of services because of a lack of professionals, institutions or certain practices. Availability can be impacted by spatial and temporal factors [23], such as the distance between the service user (in our case, the pregnant woman) and the health care facility, and the time spent waiting or traveling [24].

Appropriateness reflects the technical and professional aspects of care and their adequacy, i.e. what services are provided and how they are provided [19]. It also refers to the appropriateness of the facilities and their environmental aspects. Appropriateness of care entails two dimensions, namely clinical and social quality [23]. In maternal care, clinical quality refers to the quality of procedures and care delivered by health professionals, while social quality refers to facility maintenance, accommodation and environment [25]. In order to improve the health outcomes, healthcare must be of good quality: safe, effective, timely, efficient, equitable and people-centred. In 2016, WHO published standards for improving the quality of maternal and newborn care, which place people at the centre of care by improving both the provision of, and patients’ experience of, health care. Provision of healthcare includes evidence-based practices for routine care and management of complications, actionable information systems and functioning referral systems while experience of care includes effective communication, respect and preservation of dignity and emotional support. These factors, along with competent and motivated human resources, and essential physical resources, are a critical part of ensuring the quality of (maternal) care [26].

Affordability reflects the payments made by the service user, including various types of out-of-pocket payments but also indirect payments (e.g. travel costs) that make care less affordable and limit access to it [19]. Affordability barriers mean that even if care is available and appropriate, the childbearing woman might be unable to access it because she cannot pay for it. Out-of-pocket expenses can be classified as: formal (regulated by national legislation), quasi-formal (official charges set up by providers outside national regulation), informal (unofficial payments or gifts by the service user) and quasi-informal (e.g. medical products brought by the service user) [27]. All types of out-of-pocket payments can severely limit the ability to access maternal care [15, 28].

Approachability refers to the awareness of service availability among service users, as well as to the information distributed regarding available treatments and services [19]. It also refers to the psychological dimension of accessibility, which might be hindered by poor communication, resulting in social distance and mistrust, and even by discrimination on the side of health care staff [23]. Thus, care could be available, appropriate and affordable, but pregnant women may not use it due to the lack of information or some psychological access barriers [24].

Acceptability is determined by cultural, traditional and literacy factors that determine whether institutionalized care is accepted by individuals, as well as whether and how often care will be demanded [19, 29]. Thus, care might be available, appropriate, affordable and approachable, but not acceptable due to cultural, traditional and health literacy aspects in determining the need for institutionalised care [21]. Acceptability can also be influenced by the health care provider, such as when non-acceptance of pregnant women influences care-seeking behaviour and leads to unwillingness to seek care [30].

**Fig. 1:**
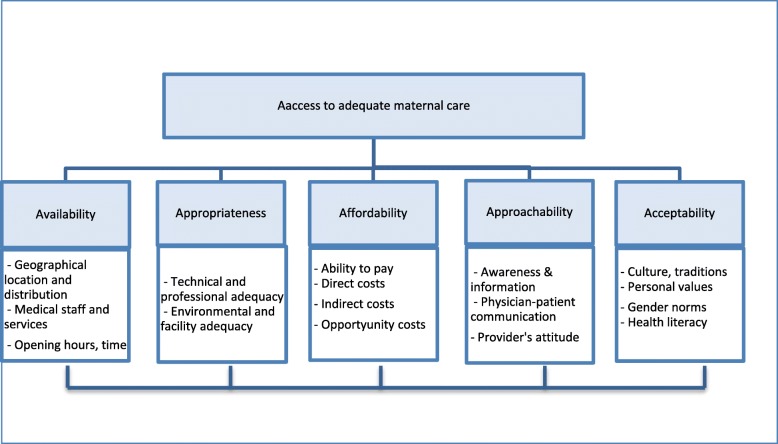
Barriers to access to adequate maternal care. Source: Authors’ compilation, based on Levesque et al. [19]

Figure 1 illustrates the operational definition of access to adequate maternal care applied in this study, based on the framework proposed by Levesque et al. and the additional considerations outlined above [19].

## Methods

This study follows an explorative qualitative approach and applies the method of directed content analysis [31] based on pre-selected themes (the five aspects of access shown in Fig. 1). Ethical approval of the study was obtained from the Bioethical Committee of the National Center for Disease Control and Public Health of Georgia prior to data collection.

The data were collected in May–June 2015 in two urban settings (Tbilisi and Kutaisi) and one rural setting (Batumi area). Tbilisi is the capital city located in the eastern part of Georgia, Kutaisi is a central region of Georgia and Batumi is a region located in south-west Georgia. Two methods of data collection were used, allowing for data triangulation. First, focus group discussions (FGDs) were carried out with women who gave birth in the preceding years, to elicit what barriers (if any) to accessing maternal care they had experienced. This was complemented by semi-structured in-depth interviews (IDIs) with decision-makers and health care professionals to gain an understanding of their opinions about access to maternal care. We then identified similarities and differences in the opinions across the three stakeholder groups. This allowed us to gain insights from multiple perspectives and to better understand the study phenomenon. It gave a voice to the experience of stakeholders and offered the opportunity to explore the depth and complexity of access to adequate maternal care in Georgia.

In total, six FGDs were conducted, two in each study setting. Each group consisted of up to 10 women who had experienced their last childbirth within the preceding four years. One group in each setting consisted of women who had one child and the other group consisted of women who had two or more children. No criteria for age or economic status were applied to enable exploring possible access barriers among all women in their reproductive age and representing diverse income groups.

The contact details of the women were obtained through hospital registries, combining the methods of purposive and convenience sampling. The women were approached by one of the researchers via phone and were asked to participate. Women who agreed to participate were asked to reach out for someone in their social network who would meet the inclusion criteria and would be willing to participate. No refusal to participate was registered. All participants were asked for their verbal informed consent to participate prior to the FGDs taking place.

The FGDs were conducted in Georgian language. An experienced discussion moderator led the discussion and two researchers participated as observers. The discussions included questions that were developed in accordance with our operational definition of access to adequate maternal care (see Fig. 1). The questions are presented in Additional file 1. To facilitate the communication, during the introduction, all women were provided with cards to write down their names. The discussion started with stating the general purpose of the FGD and establishing ground rules. After that, the topic was introduced and discussed based on the pre-selected questions. After each question, women were given time to note down their ideas, after which an open discussion took place. At the end of the discussion of each topic, the results were summarised verbally by the FGD leader and at the end of the FGD, the participating women were asked for additional comments or opinions. The discussions were recorded, transcribed and translated into English.

The IDIs followed the FGDs. Five decision-makers and four health care professionals were involved in the study. The decision-makers and health care professionals were selected through purposive and convenience sampling, based on their position and importance in the field of maternal care in Georgia. The decision-maker group was represented by two decision-makers from international institutions, namely USAID and UNICEF Georgia, one decision-maker from the National Center for Disease Control (NCDC) and two decision-makers from the Ministry of Health. The health care professional group was represented by three gynaecologists of whom two were also working at the medical university, and one member of the management team of the biggest maternity house of Tbilisi. In view of the “technocratic model of care” in place in Georgia, no midlevel providers such as midwives were included as participants in the interviews [13].

The interviews were carried out in Tbilisi and Kutaisi, while the potential participant (health care professional) from Batumi region cancelled the interview and it was not possible to find another suitable replacement in the given time. Therefore, no interviews were conducted in this region. All interviewees were asked for written informed consent prior to the interview. Two researchers participated during the interviews, one of whom led the interviews. The IDIs included questions similar to those used in the FGDs (Additional file 2). Six interviews were held in English and three in Georgian. The interviews were recorded, transcribed and, where necessary, translated into English.

As mentioned above, the data collected through the FGDs and IDIs were analysed based on the qualitative method of directed content analysis [31]. The guiding themes for the content analysis were the five aspects of access to maternal care presented in Fig. 1. The transcripts were first read to identify relevant information, which was then clustered in the five themes. The categorised information was analysed and similarities and differences across the stakeholder groups identified. Participant quotes are displayed throughout the Results section to provide a narrative presentation of key findings.

## Results

The two FGDs in Tbilisi consisted of 14 women in total, the two FGDs in the Kutaisi town of 15 women and the two FGDs in the rural Batumi region of 15 women. The women in all FGDs were aged 19–42. The IDIs with maternal care professionals (four interviews) and decision-makers (five interviews) included both men and women of diverse age groups. The results are presented below in accordance with the five access-related themes and are illustrated with quotes. The full list of quotes can be found in Additional file 3.

### Availability

Almost all women in the FGDs expressed their disappointment with the unavailability of postnatal care. This is a problem when, for example, women need support with breast-feeding or assistance with post-childbirth complications.

*‘I had problems with breastfeeding and it was a problem that there was no postnatal care which strikes most mothers’ (FGD, single child, Tbilisi).*

In the rural area of Batumi, specific services to manage complicated cases before childbirth are not available according to the participants in our FGDs. For these services, women have to have a journey to Batumi city or even to the capital, Tbilisi, which means a long-distance travel for these women to the very east of Georgia. Participants noted that these necessary services should be available closer to their home. However, even in the capital city Tbilisi, a participant noted an insufficient number of incubators and hospital beds. Due to the unavailability of hospital beds, women were discharged earlier from hospital or asked to find another institution. In addition, some women mentioned a shortage of staff (e.g. anaesthesiologists) in low capacity institutions, especially in rural areas.

Health care professionals also perceived human resources as an important problem, especially in rural areas.

*‘Some kind of services could be unavailable when needed; especially in rural areas some services are really missing and could contribute to access issues and therefore quality of health care. Human resources are an issue’ (IDI, gynaecologist, Tbilisi).*

They mentioned that sometimes pregnant women who had overcome the spatial barrier in reaching a health care provider could not access the necessary care because the specific service was not available (e.g. USG). This created another barrier or delays in health care utilisation.

Decision-makers identified the recent privatisation of the health system (which was implemented in 2007) as an additional barrier in terms of service availability, due to the freedom of providers to choose the type of services they want to provide based on their interest (thus, sometimes not providing maternal care). They also mentioned that assistance during pregnancy and the postnatal period was usually provided only by obstetricians, whereas inclusion of maternal care within family doctor services would improve accessibility. Overall, the opinions of health professionals and decision-makers regarding availability-related barriers were in line with those expressed in the FGDs.

*‘Family doctor involvement and assistance is not available for pregnant women, but would be useful to improve access (IDI, Health Ministry, Tbilisi).’*

The women in the FGDs indicated that access to maternal care was complicated for women living in rural areas, especially in high-mountain regions, due to large travel distances and a weak transportation infrastructure.

*‘Distance is an issue for women from rural areas, because in the capital the care is more adequate and modern than in rural areas (FGD, single child, Tbilisi).’*

Furthermore, the travel costs are high; roads are of poor quality and public transport, if existent, has a very poor schedule and only few destinations. In contrast to rural areas, care in the capital was reported to be more advanced and adequate. In the women’s opinion, these were the main reasons why geographical distances could lead to delayed care-seeking behaviour and poor health outcomes.

Most decision-makers and health professionals agreed with the women that rural and high-mountain populations are more likely to face access barriers and inadequate maternal care. Both groups emphasized that even though most women could access some maternal care, the problem was to access adequate care close to their place of residence. Therefore, the rural population groups are most likely to experience pregnancy-related complications and the need to travel for suitable care. The representative of the Georgian Ministry of Health also highlighted the problem of lacking transportation infrastructure and availability of care in various regions, especially in remote areas, which were important contributors to access barriers.

Only a few women from Tbilisi and Kutaisi reported not to have experienced any problems with service availability. One gynaecologist claimed that in his hospital all services were available at any time of the day. Somewhat similarly, a decision-maker stated that postnatal care officially existed in Georgia and was covered by the state, but that women had to be aware of the need for it, since it worked through self-referral. There was a gap in policy implementation, because the information on the availability of postnatal services was not reaching all providers and women in need.

All participants agreed that there were no distance-related access problems in the capital city. Participating women from Batumi region who lived in suburbs or rural areas claimed that the distance to adequate care in Batumi city was not that long, which therefore was not a barrier. None of the FGD or IDI participants reported waiting lists for maternal care. Nevertheless, a clinical manager reported that women usually preferred to receive care in the area in which they lived. However, when they needed more advanced care available in the capital, the cost was the problem, not the distance they had to travel.

### Appropriateness

Most participants stated that there were problems with the appropriateness of maternal care in Georgia. The women reported the need to search for adequate care because quality differed across the country, a city or even within an institution.

***‘*** *Everywhere the care is not of good quality; you need to search for it’ (FGD, multiple children, Tbilisi).*

However, they noted that they were free to choose a physician, facility and the care they preferred, resulting in the need to find the best options for them. The adequacy of maternal care was also influenced by the conditions in the facilities, which were reported to have improved since the privatisation of health care. However, the participants still reported the existence of old, non-renovated buildings, unhygienic facilities and bathrooms that were out of order.

Despite the existing inadequacies in maternal care that, according to an advisor to the Ministry of Health, were more prevalent in smaller towns or rural areas, decision-makers argued that this did not create any barriers for women seeking care, as they were free to choose where to go. They claimed that inadequate maternal care in some facilities should not result in postponed or unused maternal care in a city. However, they conceded that quality of care might influence health outcomes. Health professionals added that inadequate antenatal care contributes to the high MMR in Georgia. Part of the problem was reported to be underqualified staff.

*‘In Georgia, maternal mortality is high due to low quality of antenatal care. Problems are not identified in time, because of underqualified staff’ (IDI, gynaecologist, Tbilisi).*

In contrast, other participants suggested that the adequacy of maternal care in Georgia was not a barrier to access. Some women reported that facility conditions had been critical in the past, but that these had generally improved and were not a reason for not using maternal care services. Furthermore, some women highlighted that the free choice of health care providers and facilities meant that adequacy of care should not be a barrier. Some women also shared positive experiences, reporting that they were highly satisfied with the attitudes and conditions of the care they had received. Women often relied on advice from family or friends to avoid poor quality experiences.

*‘I knew a good doctor through friends and I was also very happy with the services, [I did not have any] negative experiences of quality or attitude’ (FGD, single child, Tbilisi).*

Decision-makers agreed that there should be no major adequacy problems due to the lack of infrastructure. However, they pointed out that what happened during antenatal visits was difficult to measure and that adequacy problems might be involved. Decision-makers also agreed with the women that, due to the free choice of care, a woman could “shop” for quality.

*‘Women that die have no attendance issues, the problem can be found in the quality of care and the poor recognition of health complications. It is important to know where to go for good care and not everyone knows those things’ (IDI, USAID, Tbilisi).*

### Affordability

The majority of the women in the FGDs indicated that the financial aspect was an important barrier to accessing adequate maternal care. According to these respondents, the state programs covered only the basic needs of women, but all additional antenatal visits, tests and medications had to be paid out-of-pocket. Women claimed that maternal care could become a real burden for pregnancies with complications and for mothers from low-income families, especially those living in rural areas.

*‘I needed an extra test due to my high-risk pregnancy that was expensive. I had to pay out of pocket and I needed support from family, otherwise it was not possible (FGD, multiple children, Tbilisi).’*

The rural population was seen as having limited possibilities to earn an adequate income. Therefore, even indirect costs, such as extra traveling costs of 20 lari (8 Euros) to reach the health care facility could be a financial burden. However, family members were reported to always support each other, which allowed a number of women to afford the necessary maternal care that otherwise in many cases would not be affordable. A woman in Tbilisi reported that due to the high costs of an unexpected caesarean section, she had to change her facility to a cheaper one, because she could not afford it. Furthermore, another woman from Tbilisi reported that she was not able to afford necessary laboratory tests that costed 2000 lari (800 Euros). During the FGDs, affordability figured as an important barrier to accessing adequate care, which in some cases made women postpone necessary care until the issues became more serious and often even dangerous.

Decision-makers also viewed out-of-pocket payments, especially for high-risk pregnancies, as an important burden for vulnerable population groups.

*‘Providers are charging for additional visits and doing tests that add costs. It can be a burden for vulnerable population groups, such as the poor’ (IDI, USAID).*

The poor and those living in rural areas were more affected due to their low income and extra travel expenses. Furthermore, decision-makers claimed that pharmaceutical costs were in most cases paid 100% out-of-pocket and were not affordable for everyone. According to the IDIs, even in uncomplicated pregnancies, every additional antenatal visit, test or medical intervention that was not covered by the state programs, had to be paid out-of-pocket. Consequently, women were forced to postpone care and this might endanger their health and ultimately increase health care costs, if care became unavoidable. A clinical manager from one of the biggest maternity houses in Georgia pointed out that some women had to search for another, cheaper institution, because they could not afford the care in this clinic.

*‘In my facility, the price is high and some women cannot access the care here and have to go somewhere else. Universal coverage only covers basic needs’ (IDI, maternity house manager, Tbilisi).*

Despite a number of participants reporting affordability problems, several stated the opposite. A woman in Tbilisi spent around 600 Euros during her pregnancy, but said she was prepared to pay this price. She added that women who were able to pay more could choose more luxurious care options if they preferred. Some women argued that having a private health insurance helped in covering maternal care costs. However, only those with a sufficient regular income could afford and purchase the insurance packages.

Some women in the FGDs stated that the state program for maternal care worked quite well and helped them to cover the costs that arose from complications or the necessity to be transferred to another institution. Some women reported that out-of-pocket payments often seemed high, but that this was not a reason not to use care. One woman added that childbirth is a happy event for which a woman is happy to spend money.

*‘My husband and me are working, therefore we do not face financial barriers (FGD, single child, Kutaisi).’*

A number of stakeholders in the IDIs agreed that every woman received basic care, so the lack of financial resources should not constitute an access barrier. They maintained that, since the new state program has been implemented, affordability has at least become a much smaller problem. At the same time, the program was a stimulus to seek care on time in order to be covered.

*‘We have the law that if a woman is delaying her care then the state program is not supporting her anymore and she has to pay out of pocket, and in that way, she is stimulated to seek care on time’ (IDI gynaecologist, Tbilisi).*

However, these opinions clashed with those situations where women lacked money to purchase extra maternal care services or their condition fell outside the state program. Overall, the opinions of decision-makers and care providers were divided over the existence of affordability problems.

All FGD participants denied the presence of informal payments, arguing that, since the privatization of health care, all payments in Georgia have been official. However, a number of women reported that they showed voluntary gratitude to medical staff by giving gifts in kind.

### Approachability and acceptability

In almost all FGDs, women reported that they had experienced inappropriate attitudes from healthcare staff and that they would not seek care in the same institution again.

*‘I experienced poor attitudes and ignorance by healthcare providers’ (FGD, multiple children, Tbilisi).*

Decision-makers also reported problematic attitudes and poor communication by healthcare workers, which they believed to influence the quality of care and, ultimately, the health outcomes of women.

*‘Attitudes and responsiveness of healthcare providers, including the consultation time, is worrisome, which influences the delay in care and safety of women’ (IDI, NCDC, Tbilisi).*

A gynaecologist agreed that, despite improvements, problematic attitudes from providers exist, especially in rural areas. Women were then not free to share their experiences or problems, further impinging on quality of care.

*‘Poor communication can influence quality of care and when women are unhappy due to poor communication they are not able to share their experiences or problems’ (IDI, gynaecologist, Tbilisi).*

Approachability and acceptability problems with regards to information and communication were thus interrelated. The most important problems reported in the FGDs were related to a lack of information about maternal care, which resulted in delayed or irregular visits. Women reported that they sometimes did not understand what doctors meant and that they felt confused by all the medical terms. In addition to information acquired within social networks, the women expressed the wish to receive more education from healthcare professionals, which would help them throughout the pregnancy and during the postnatal period. For instance, women said they were uninformed about childbirth, breastfeeding and different programs that covered high-risk pregnancies.

*‘I don’t know about programs covering high-risk women and if we are not informed about different programs we don’t know what services we can have’ (FGD, multiple children, Batumi).*

Healthcare professionals and decision-makers agreed that pregnant women, especially in rural areas, lacked information and education. They also agreed that this information barrier was causing care to be postponed, with potential complications. If a woman was not aware of her health condition, she was less likely to take any preventive measures or to engage in pro-active behaviour. The representative of the Ministry of Health explained that in the Georgian culture there was a general fear and poor trust in medical help. Thus, people had little understanding of the benefits of prevention, which could contribute to poor health outcomes.

All FGD participants reported that they did not see any religious or cultural barriers to accessing maternal care and they did not think there were women who would not feel the need for professional care during pregnancy, childbirth and the postnatal period.

*‘I haven’t heard of any cultural or religious reasons that could act as barriers to accessing maternal care, at least not for the Georgian population’ (FGD, single child, Tbilisi).*

Despite the large information insufficiencies, a few women argued that they were very well informed about their pregnancy and the importance of antenatal visits. One mother reported a very positive experience with her physician who gave her daily check-up calls to advise on the use of medications. Women from Batumi said they had no complaints about inappropriate attitudes by health care providers.

*‘We are generally satisfied with the attitudes we encountered from our doctors’ (FGD, multiple children, Batumi).*

One gynaecologist argued that the lack of information was unusual, since women shared information within their social networks. Overall, health professionals and decision-makers agreed that in general women accepted maternal care, but they pointed out that it was not always timely or adequate.

*‘Even if the women are poorly informed during the antenatal and postnatal period, insufficient information is not a barrier to reject the institutionalized maternal care services’ (IDI, health ministry, Tbilisi).’*

None of the IDI participants thought that women were informed enough about maternal care. The lack of information seemed to increase the risk of delayed and insufficient care. However, none of the participants identified other factors, such as culture, religion or gender roles, as constituting barriers to accessing adequate maternal care.

## Discussion

The stakeholder views on access to adequate maternal care in Georgia reported above indicate several important problems that need to be addressed in future reforms. These problems are related to maternal care standards, inequalities across population groups and maternal care financing.

### Maternal care standards

Our findings suggest that the standards of maternal care provision in Georgia are a major concern. This involves gaps in clinical quality and staff skills, as well as inadequate attitudes by health workers and poor communication between women and health professionals. These findings are in line with what has been documented in the literature, including the so-called “technocratic model” [13, 14], mentioned in the Background section. This model undermines the needs of women, their babies and families, which results in women not being in the centre of care. The model explains the highly-specialised model of care and the exclusion of midlevel providers such as midwives [15].

Quality standards of medical care and medical technologies in Georgia are not regulated by law and service providers, including those in the private sector, are responsible for setting their own indicators [4]. However, it should not be overlooked that many women seem to comply with two international standards: four antenatal care visits and skilled delivery. Maternal care providers who are well known for their good services are reported to increase their prices, but more expensive services do not always secure higher quality standards [4, 7]. The existing evidence supports our participants’ arguments that medical staff have poor professional standards due to the lack of continuous education programs in the Georgian health system [32]. The low salaries of health care professionals also demotivate them to perform adequately [12].

The literature also supports the finding from our study that the information that women in Georgia receive about the importance of adequate maternal care is insufficient, especially among those with lower levels of education and populations living in rural areas. This may lead to delayed care-seeking behaviour and overlooked complications, which may result in poor health outcomes [7, 33]. None of the women and only one decision-maker in our study had information about the existence of postnatal services. In the literature, it is reported that only about 20% of women who had given childbirth in Georgia use postnatal services. There might be a problem with the implementation of maternal care programs and the dissemination of related information [4, 7] Poor health literacy of maternal care users is aggravated by problematic attitudes of health care providers. Due to disrespectful behaviour and miscommunication of some maternal care providers, women may tend to mistrust health care professionals and be unwilling to share their thoughts and preferences for care. This communication barrier might be another reason that prevents women from receiving adequate care and early detection of complications.

Nevertheless, throughout the findings there are also indications of new and positive developments. Thus, overall there are mixed findings on quality of care - there are improvements (e.g. improved coverage of care, increased use of maternal care services, reduction of informal payments, free choice of institution and healthcare provider, improvements in the conditions of facilities and also some improvements in the attitudes of healthcare staff), in addition to the challenges outlined above.

### Rural, mountain and minority population groups

According to our study, geographical barriers to access maternal care only exist in rural and mountainous areas. The distance to urban maternal care facilities that provide the necessary care can be a problem for people living in these areas due to the weak infrastructure (poor roads and weak transportation) combined with financial hardships [7, 32]. Such rural and mountainous areas suffer from a shortage of medical staff and adequate equipment, and only limited health care services are available. Even though Georgia has an oversupply of medical doctors, there is no incentive for them to work in the distant rural areas. Many of those who choose this option lack skills to provide adequate support to women and to manage pregnancy complications [4, 12, 32].

It has been suggested that many maternal care services could be handled by family doctors to improve geographical access and efficiency. Currently, these providers are not involved in the provision of maternal care [4].

Georgians do not seem to have problems with utilizing maternal care, at least as far as antenatal care and childbirth are concerned. This is supported by official statistics showing almost 100% use of antenatal care and professional birth attendance during childbirth [7, 34]. However, the literature also suggests that the use of maternal care services might be an issue for women from other cultures (ethnic minorities) [7]. Minority groups were not among the study participants and therefore this remains an open question.

### Maternal care financing

Both in rural and urban areas, good quality medical equipment seems to be frequently unavailable. This might be due to the underfunding of the healthcare sector and the inefficient use of resources [35]. Despite improvements in the coverage of maternal care, affordability remains an access barrier. Poorer population groups are financially unprotected from the high out-of-pocket payments in the healthcare sector and therefore suffer from the high costs of accessing adequate health care, including maternal care services [4, 32, 33]. In particular, all additional antenatal care visits (beyond the four visits covered), all medical tests, medications and extra child-birth costs are being paid out of pocket. Vertical programs (also known as stand-alone programs or vertical approaches) in Georgia cover cases with serious complications, which may encourage women to postpone care-seeking behaviour [7, 36].

In 2016, WHO came up with new recommendations on antenatal care for a positive pregnancy experience, which include antenatal care models with a minimum of eight visits. The eight-visit plan aims to reduce maternal mortality and improve women’s experience of care [37]. This plan could potentially be realised in Georgia as many women in Georgia already receive nearly ten visits following the physician’s request [36]. However, the current model is based on the previous four-visits standard publicly provided free-of-charge. As mentioned above, after this number of visits women pay for every additional visit out-of-pocket and these payments remain a considerable burden for households due to the additional antenatal care visits and fees for “personal doctors”. The implementation of the new WHO standard would mean an extension of a basic antenatal care package from four to eight visits, but it is not certain how feasible it will be for a resource-constrained country such as Georgia [36].

Those participants in our study who claim that adequate maternal care in Georgia is affordable might see it from their perspective only and overlook the situation of the very limited resources some women have. Women who are satisfied with the amount of out-of-pocket payments during the maternal period most often have a stable income that allows them to afford the care they need [32]. Overall, informal payments do not seem to be contributing to barriers to accessing adequate care.

### Relevance of the study to other settings

Although our study focuses on Georgia, given the general theoretical framework applied, it may be relevant to researchers and decision-makers in other countries as well. Our results show the importance of micro-level indicators, such as disrespectful behaviour by health professionals and their attitudes towards pregnant women, as well as women’s trust in maternal care providers and care acceptability. In addition to existing macro-level indicators, e.g. the numbers of providers and facilities in the country, such micro-level indicators have to be taken into account for a comprehensive evaluation of the provision of maternal care. This is especially relevant for countries in Eastern Europe where maternal care problems might remain concealed by comparatively good macro-level indicators [22, 38, 39].

### Strengths and limitations of the study

Our study has several strengths and limitations. The qualitative design allowed us to capture a rich and detailed picture of the researched topic. However, the small number of participants and settings covered makes it difficult to generalize findings. The quality of the data collection and analysis is highly dependent on the researcher’s skills. This bias was mitigated to some degree by involving two researchers during the interviews and focus-group discussions, and an experienced discussion moderator. We also acknowledge possible recall bias and the potential for socially-desirable answers in both, the focus-group discussions and the interviews. A part of the interviews was held in English, which might have created a language barrier for some respondents. The rest of the interviews and all of the focus-group discussions were transcribed in Georgian and then translated into English, due to which some nuances might have been lost. An important advantage of our study was the possibility to triangulate the opinions of the three stakeholder groups, which allowed for a broader view on access-related problems in maternal care in Georgia to emerge, as well as for the validation of findings.

## Conclusions

The study presented in this paper has explored stakeholder views on access to adequate maternal care in Georgia. Based on Levesque et al. [19], we distinguished five groups of access domains related to availability, adequacy, affordability, acceptability and approachability of maternal care. We used these domains to examine the views of maternal care stakeholders and to identify key barriers to accessing adequate maternal care. Our findings indicate the existence of a number of barriers, including inadequate quality standards, low government funding, and gaps in coverage for specific population groups. These shortcomings in maternal care in Georgia may help to explain the high maternal mortality in the country.

Problems in maternal care provision involve the lack of equipment, human resources and evidence-based treatment. Geographical distance is also problematic for rural and high-mountain population groups due to care being concentrated in the capital city, weak transport infrastructure and high traveling costs. In addition to this, Georgian women have to carefully select an available provider of care to avoid problems such as inadequate attitudes, poor clinical quality or appalling conditions at the maternal care institution. This will be challenging even for well-off and better informed and educated women, but even more so for women with fewer resources and provider options. Gaps in the knowledge and skills of health professionals, the low health literacy of women and the resulting communication problems may prevent women from receiving high-quality care, which may contribute to poor health outcomes.

These findings imply that micro-level indicators, e.g. disrespectful behaviour by health professionals, their attitudes towards pregnant women, women’s trust in maternal care providers and care acceptability, should be taken into account when assessing maternal care provision in Georgia. Such micro-level indicators should complement the existing macro-level indicators for a comprehensive evaluation of maternal care, both in Georgia and in other countries.

## Additional files

Additional file 1: Key question included in the focus group discussions. This file contains a list of questions used during focus group discussions with mothers. (DOCX 15 kb)

Additional file 2: Key question included in the in-depth interviews. This file contains a list of questions used during in-depth interviews with healthcare professionals and decision makers. (DOCX 15 kb)

Additional file 3: Boxes with participant quotes. This file contains the full list of study participant quotes in accordance to the five access-related themes. (DOCX 22 kb)

## Abbreviations

FGDs: Focus group discussions
IDIs: In-depth interviews
MMR: Maternal mortality rate
NCDC: National Center for Disease Control and Public Health
