# Supplementary material for: Barriers to accessing adequate maternal care in Georgia: a qualitative study
Source: BMC Health Serv Res. 2018 Aug 13;18:631. doi: 10.1186/s12913-018-3432-z (PMC6090778; doi:10.1186/s12913-018-3432-z)
Supplement: Supplementary file 1 — Key question included in the focus group discussions. This file contains a list of questions used during focus group discussions with mothers. (DOCX 15 kb) [file 12913_2018_3432_MOESM1_ESM.docx]

**Additional file 1**

**KEY QUESTION INCLUDED IN THE FOCUS GROUP DISCUSSIONS**

1. Do you think that the payments for maternal care were a problem to access/use certain services?
2. Did you ever have to pay for maternal care unofficially in cash or kind?

− If yes, what was the reason for it? (e.g. gratitude, ensuring quality, it is widely accepted method)

1. In your opinion, what limits quality of antenatal, postnatal and delivery care services in Georgia to you and all women irrespective of their socio-economic status and health conditions?
2. Do you think that the limited quality of maternal healthcare services was a barrier to seek for the care (e.g. poor attitude, conditions in healthcare unit, treatment itself)?
3. Did you experience any maternal care services being not available in the area you live (either not existent or shortage of availability) during antenatal, delivery or post-natal period?

− Was that a barrier to seek for the health service?

1. Did you experience any issues to access maternal care services during antenatal, delivery or post-natal period in terms of distance or time?

− Was that a barrier to seek for the necessary health service?

1. Do you perceive a need to receive maternal healthcare services during all the 3 phases – pre/postnatal and delivery?
2. Could you tell whether there are any maternal care services that you think are not necessary/important?
   - Is that a reason you did not seek for them?
3. Did you feel like you were missing information on the use of maternal services?
4. Where there any other reasons that held you back from or were a barrier of using maternal care services provided by healthcare professional (e.g. culture, religion, gender relationship in family)?
