# Supplementary material for: Barriers to accessing adequate maternal care in Georgia: a qualitative study
Source: BMC Health Serv Res. 2018 Aug 13;18:631. doi: 10.1186/s12913-018-3432-z (PMC6090778; doi:10.1186/s12913-018-3432-z)
Supplement: Supplementary file 2 — Key question included in the in-depth interviews. This file contains a list of questions used during in-depth interviews with healthcare professionals and decision makers. (DOCX 15 kb) [file 12913_2018_3432_MOESM2_ESM.docx]

**Additional file 2**

**KEY QUESTION INCLUDED IN THE IN-DEPTH INTERVIEWS**

1. Do you think that the payments for maternal care are a problem to access/use certain services?
2. Have you experiences that maternal care services are handled unofficially in cash or kind?
3. If yes, what in your opinion is the reason for it? (e.g. gratitude, ensuring quality, it is widely accepted method
4. In your opinion, what limits adequacy of antenatal, postnatal and delivery care services in Georgia to all women irrespective of their socio-economic status and health conditions?
5. Do you think that the limited quality of maternal healthcare services was a barrier to seek for the care (e.g. poor attitude, conditions in healthcare unit, treatment itself)? Could you explain why?
6. In your opinion, what are the main barriers to seek for the necessary maternal healthcare service in terms of space and time? Please explain your opinion.
7. Are any maternal care services being not available (either not existent or shortage of availability) during antenatal, delivery or post-natal period?
   - Is the availability a barrier to seek for the health service?
8. Is there diversity of perceived need of maternal healthcare services among different women during all the 3 phases – pre/postnatal and delivery?
9. Could you tell whether there are any maternal care services that some women might perceive as not necessary/important?
   - Is that a reason not to seek for them?
10. Do you think some women are missing information on the use of maternal services?
