# Supplementary material for: Barriers to accessing adequate maternal care in Georgia: a qualitative study
Source: BMC Health Serv Res. 2018 Aug 13;18:631. doi: 10.1186/s12913-018-3432-z (PMC6090778; doi:10.1186/s12913-018-3432-z)
Supplement: Supplementary file 3 — Boxes with participant quotes. This file contains the full list of study participant quotes in accordance to the five access-related themes. (DOCX 22 kb) [file 12913_2018_3432_MOESM3_ESM.docx]

**Additional file 3**

**BOXES WITH PARTICIPANT QUOTES**

***Box 1 Statements that indicate availability problems***

| **Women**  ‘Not enough incubators and beds in some hospitals even in capital city. Postnatal care does not exist, which is an issue.’ (FGD, multiple children, Tbilisi).  ‘I had problems with breastfeeding and it was a problem that there was no postnatal care which strikes most mothers.’ (FGD, single child, Tbilisi).  ‘More advanced services are available only in bigger cities.’ (FGD, single child, Batumi).  ‘Availability of anaesthesiologist was an issue.’ (FGD, single child, Kutaisi).  ‘Antenatal care is difficult, because we are in a rural area. We cannot always seek care in Batumi or Tbilisi city.’ (FGD, multiple children, Batumi).  ‘Distance is an issue for women from rural areas, because in the capital the care is more adequate and modern than in rural areas.’ (FGD, single child, Tbilisi).  **Health professionals**  ‘Some kind of services could be unavailable when needed; especially in rural areas some services are really missing and could contribute to access issues and therefore quality of health care. Human resources are an issue. (IDI, gynaecologist, Tbilisi)  ‘There are issues in accessing care in high-mountain areas. The rest receive some care, just depends on what quality.’ (IDI, gynaecologist, Tbilisi).  ‘Georgia has a three-delay morale [decision to seek care, reaching care, receiving adequate care]. Once the decision [to seek care] has been made, that there is a spatial barrier, more prevalent in rural and mountain areas.’ (IDI, gynaecologist, Tbilisi).  ‘The third delay is when you have overcome the decision and spatial delays, but the hospital or the type of care you need is not there which happens in Georgia’ (IDI, gynaecologist, Tbilisi).  **Decision-makers**  ‘Privatization creates an access issue because the providers can decide what services they want to provide.’ (IDI, ministry advisor, Tbilisi).  ‘Family doctor involvement and assistance is not available for pregnant women, but would be useful to improve access.’ (IDI, Health Ministry, Tbilisi)  ‘There is a lack of anaesthesiologists and C-section provision in low-volume facilities’ (IDI, USAID, Tbilisi)  ‘There is no postnatal care available, but it should be mandatory since it contributes to maternal and child health outcomes.’ (IDI, NCDC, Tbilisi)  ‘No geographical access issue per se, but rather quality access issue with geographical differences in case of complications.’ (IDI, USAID, Tbilisi)  High-mountain and rural areas have issues with substandard care.’ (IDI, NCDC, Tbilisi)  ‘Travel distance, especially in rural areas, is coupled with financial problems.’ (IDI, UNICEF, Tbilisi)  ‘Challenge of transportation of new-borns and referrals in Georgia creates access barrier.’ (IDI, ministry advisor, Tbilisi).  ‘Healthcare regionalization instead of the existing centralized system would improve accessibility to good maternal services’ (IDI, Health Ministry, Tbilisi) |
| --- |

***Box 2 Statements that indicate appropriateness barriers***

| **Women**  **‘**Everywhere the care is not of good quality; you need to search for it.’ (FGD, multiple children, Tbilisi).  ‘Adequacy of care is not unified in the entire country.’ (FGD, single child, Tbilisi)  ‘Poor conditions at the facility (visual, hygiene issues). When I needed help, nobody came to help me and my husband had to manage it.’ (FGD, multiple children, Kutaisi).  **Health professionals**  ‘The women are accessing antenatal care, but what happens during those visits is an issue.’ (IDI, gynaecologist, Tbilisi).  ‘In Georgia, maternal mortality is high due to low quality of antenatal care. Problems are not identified in time, because of underqualified staff’ (IDI, gynaecologist, Tbilisi)  **Decision-makers**  ‘In regions, maybe it is more of a problem.’ (IDI, Ministry Advisor, Tbilisi).  ‘Direct effects on quality are services clearly lacking standards and there is the tendency from providers to perform unnecessary treatments or tests and prescribe unnecessary drugs. Women that die have no attendance issues, the problem can be found in the quality of care and the poor recognition of health complications. It is important to know where to go for good care and not everyone knows those things.’ (IDI, USAID, Tbilisi) |
| --- |

***Box 3 Statements that suggest there are no appropriateness barriers***

| **Women**  ‘Previously, there were condition problems in the facility, but they have improved a lot. Since women have free choice where to go and choose the quality they like, it does not contribute to access barriers.’ (FGD, multiple children, Tbilisi).  ‘I was satisfied with the good attitudes from doctors and the proper management of my pregnancy that involved complications.’ (FGD, multiple children, Tbilisi).  ‘I knew a good doctor through friends and I was also very happy with the services, [I did not make any] negative experiences of quality or attitude.’ (FGD, single child, Tbilisi).  ‘We are very satisfied with the quality and the attitudes and there is no such access barrier.’ (FGD, multiple children, Batumi).  **Health professionals**  ‘When a patient is coming to me, I am not letting her go until she is well informed and understands everything. If this person is not satisfied with the quality today she will not come back tomorrow.’ (IDI, gynecologist, Kutaisi).  ‘The quality is not universal in the country, but you can choose the doctors and facilities for quality reasons.’ (IDI, maternity house manager, Tbilisi). |
| --- |

| **Women**  ‘I needed an extra test due to my high-risk pregnancy that was expensive. I had to pay out of pocket and I needed support from family, otherwise it was not possible.’ (FGD, multiple children, Tbilisi).  ‘I needed C-section that should be paid out of pocket and therefore had to change for a cheaper facility.’ (FGD, multiple children, Tbilisi).  ‘I had complications and I had to pay out of pocket, they [the state] cover expenses only when it gets extremely dangerous. This is a real access issue for the needed care and makes people to postpone the care until it gets even more serious.’ (FGD, multiple children, Tbilisi)  ‘Financial barrier in rural areas is a very high barrier to access care (even 8 Euros). Family will always pay for you in Georgia; otherwise you could not access the care you need.’ (FGD, multiple children, Tbilisi).  ‘I needed a genetic test which costed 800 Euros and I could not do it because it was too much - finance is the access issue for good care.’ (FGD, single child, Tbilisi)  ‘Pharmaceutical costs were too high for me’ (FGD, multiple children, Kutaisi).  **Health professionals**  ‘The financial aspect is very important in Georgia. Salaries are too low, especially in rural areas, and that is the main reason people are postponing care’. (IDI, gynaecologist, Kutaisi).  ‘In my facility, the price is high and some women cannot access the care here and have to go somewhere else. Universal coverage only covers basic needs.’ (IDI, maternity house manager, Tbilisi)  **Decision-makers**  ‘Providers are charging for additional visits and doing tests that add costs. It can be a burden for vulnerable population groups, such as the poor.’ (IDI, USAID).  ‘Prescribed medicine could be a barrier for some women.’ (IDI, NCDC director).  ‘There are some population groups that have covered pharmaceuticals, but the amount is very limited, which creates an access problem.’ (Health Ministry, Tbilisi).  ‘Access to maternal care can be a very big burden for families in care of complicated cases and when additional antenatal visits are needed.’ (IDI, Health Ministry, Tbilisi). |
| --- |

***Box 4 Statements that indicate affordability problems***

***Box 5 Statements that indicate there are no affordability problems***

| **Women**  ‘I paid 600 Euros and I was ready for the payment and I am happy with it.’ (FGD, multiple children, Tbilisi)  ‘I had private insurance and it helped me to pay everything, except pharmaceuticals.’ (FGD, single child, Tbilisi)  ‘I had a complicated case and I was transferred to Tbilisi region, but the state was covering that completely. This region is well covered by state programs’. (FGD, multiple children, Batumi).  ‘It is not often problematic to pay, because it is little we have to contribute and we were already financially prepared.’ (FGD, single child, Batumi).  ‘My husband and me are working, therefore we do not face financial barriers’. (FGD, single child, Kutaisi).  ‘Getting a child is such a happy event that you forget about the costs attached.’ (FGD, multiple children, Kutaisi).  **Health professionals**  ‘In general, the government arrangements for pregnant women do a good job. Every woman receives care in 11 weeks of pregnancy, so payment should not be the reason for not accessing care.’ (IDI, gynaecologist, Tbilisi).  ‘I don’t think so [that there are affordability barriers]. We have the law that if a woman is delaying her care then the state program is not supporting her anymore and she has to pay out of pocket, and in that way, she is stimulated to seek care on time.’ (IDI gynaecologist, Tbilisi).  Decision-makers  ‘Accessibility has increased since the universal coverage and affordability is an issue to a lesser extent nowadays.’ (IDI, Health Ministry, Tbilisi). |
| --- |

***Box 6 Statements that indicate approachability problems***

| **Women**  ‘I experienced poor attitudes and ignorance by healthcare providers.’ (FGD, multiple children, Tbilisi).  ‘I had some problems and I experienced attitude issues from nurses and doctors.’ (FGD, multiple children, Tbilisi).  ‘My care was delayed while being in hospital due to ignorant attitudes from healthcare workers when I called them.’ (FGD, multiple children, Tbilisi)  ‘We sometimes do not understand what doctors mean and have the impression they only want to make their patients confused.’ (FGD, multiple children, Tbilisi)  ‘There were poor attitudes in my clinic when I needed spinal anaesthesia and I would not go there again.’ (FGD, single child, Tbilisi)  ‘We experienced poor attitudes from medical staff.’ (FGD, multiple children, Kutaisi).  ‘There is an educational problem for women related to maternal care use and importance. Also, primary care gynaecologists lack information how to handle consultations.’ (FGD, single child, Tbilisi).  ‘I don’t know about programs covering high-risk women and if we are not informed about different programs we don’t know what services we can have.’ (FGD, multiple children, Batumi).  ‘We had little information about childbirth and breastfeeding, and we only have information from peers and family.’ (FGD, single child, Batumi)  **Health professionals**  ‘Poor communication can influence quality of care and when women are unhappy due to poor communication they are not able to share their experiences or problems. Provider attitudes have improved, but are still the most problematic in rural areas’ (IDI, gynaecologist, Tbilisi)  ‘Yes, it is a problem, especially in rural areas, and their antenatal care-seeking behaviour is delayed. They are missing information about the importance of it.’ (IDI, gynaecologist, Tbilisi)  **Decision-makers**  ‘Due to the lack of knowledge, they are often waiting until a critical point, hesitating to go to a doctor and hoping that everything will be OK rather than preventing problems or seeking care at an early stage.’ (IDI, UNICEF, Tbilisi).  ‘Generally, there is in the country some kind of fear to go to the doctor, because of low trust. People have a problem to understand that prevention is better than cure. Population awareness in the country of what is good quality care is generally low’ (IDI, ministry advisor, Tbilisi.)  ‘Lack of information and education (especially in rural areas) is a barrier to seek maternal care sufficiently and on time.’ (IDI, NCDC, Tbilisi)  ‘There could be problematic attitudes from providers and they can influence health outcomes, but they are no reason for not seeking maternal care.’ (IDI, UNICEF, Tbilisi).  ‘Attitudes and responsiveness of healthcare providers, including the consultation time, is worrisome, which influences the delay in care and safety of women.’ (IDI, NCDC, Tbilisi) |
| --- |

***Box 7 Statements that indicate there are no approachability problems***

| **Women**  ‘I knew a good doctor; all my family members went there and I did not experience miscommunication. After I had to start taking medication, doctor checked on me every day and I was very happy with this attitude.’ (FGD, single child, Tbilisi)  ‘I knew everything about the antenatal visits.’ (FGD, multiple children, Batumi).  ‘We all thought that care during antenatal and postnatal period is necessary, but postnatal care was not available.’ (FGD, multiple children, Tbilisi).  ‘I haven’t heard of any cultural or religious reasons that could act as barriers to accessing maternal care, at least not for the Georgian population.’ (FGD, single child, Tbilisi).  ‘We are generally satisfied with the attitudes we encountered from our doctors.’ (FGD, multiple children, Batumi)  **Health professionals**  ‘Women don’t have problems with poor attitudes, if they are not satisfied, they will go elsewhere. Lacking information is unusual. Ladies are talking with each other. Before coming to receive care, they are already informed that they need care and at least eight visits.’ (IDI, gynaecologist, Kutaisi).  No cultural or religious barriers for institutionalized care. Here natural childbirths are not happening, only in maternity wards.’ (IDI, gynaecologist, Tbilisi)  ‘All women in Georgia perceive the need to use maternal care sooner or later, and furthermore childbirths are institutionalised.’ (IDI, gynaecologist, Tbilisi).  **Decision-makers**  ‘Even if the women are poorly informed during the antenatal and postnatal period, insufficient information is not a barrier to reject the institutionalized maternal care services. Sooner or later the women do use them.’ (IDI, health ministry, Tbilisi). |
| --- |
